# Difference between Chitosan Hydrogels via Alkaline and Acidic Solvent Systems

**DOI:** 10.1038/srep36053

**Published:** 2016-10-27

**Authors:** Jingyi Nie, Zhengke Wang, Qiaoling Hu

**Affiliations:** 1 MoE Key Laboratory of Macromolecular Synthesis and Functionalization, Department of Polymer Science and Engineering, Zhejiang University, Hangzhou, China; 2 Key Laboratory of Adsorption and Separation Materials & Technologies of Zhejiang Province, Hangzhou, China

## Abstract

Chitosan (CS) has generated considerable interest for its desirable properties and wide applications. Hydrogel has been proven to be a major and vital form in the applications of CS materials. Among various types of CS hydrogels, physical cross-linked CS hydrogels are popular, because they avoided the potential toxicity and sacrifice of intrinsic properties caused by cross-linking or reinforcements. Alkaline solvent system and acidic solvent system are two important solvent systems for the preparation of physical cross-linked CS hydrogels, and also lay the foundations of CS hydrogel-based materials in many aspects. As members of physical cross-linked CS hydrogels, gel material via alkaline solvent system showed significant differences from that via acidic solvent system, but the reasons behind are still unexplored. In the present work, we studied the difference between CS hydrogel via alkaline system and acidic system, in terms of gelation process, hydrogel structure and mechanical property. *In-situ*/pseudo *in-situ* studies were carried out, including fluorescent imaging of gelation process, which provided dynamic visualization. Finally, the reasons behind the differences were explained, accompanied by the discussion about design strategy based on gelation behavior of the two systems.

Polysaccharide has received tremendous popularity as useful and renewable resource, providing opportunity in sustainable development^1^. Chitosan (CS) is a polysaccharide obtained by the deacetylation of chitin, which generated considerable interest. The potential of CS has been widely recognized due to its intrinsic properties: biocompatibility, biodegradability, biological activity, and abundance in nature^2^,^3^, which enabled a wide range of applications of CS materials, such as in food industry, water treatment, tissue engineering, wound dressing, and bone fracture internal fixation^4^,^5^,^6^,^7^,^8^,^9^. CS hydrogel is a major and vital branch in the utilization CS material. Among CS hydrogels fabricated by various techniques, physical cross-linked CS hydrogels are popular^10^, because they avoided the potential toxicity and the sacrifice of intrinsic properties caused by cross-linking or reinforcements^11^ The basic preparation of a CS hydrogel with physical cross-links is to solubilize the macromolecules in an acidic aqueous solution (referred to as acidic system). To facilitate the exploit of polysaccharide resource, a new solvent system for CS had been developed, *i.e.* the alkali metal hydroxide-urea aqueous solution (referred to as alkaline system)^12^. Besides the preparation of physical cross-linked hydrogel, these systems lay the foundations of CS material preparation in many aspects, including fabrication of CS hydrogel with sophisticated organization^5^,^13^, and preparation of CS based composite materials^14^,^15^,^16^,^17^,^18^. The most intriguing thing is, although CS hydrogels via alkaline and acidic systems are both physical cross-linked, there exists significant differences between them, especially in mechanical properties. It has been reported that CS hydrogel via alkaline system showed great improvement in hardness, strength and toughness^19^, but the fundamental reasons still remain to be explored. For the control of hydrogel structure and design of novel CS hydrogel material, it is of academic and application value to understand the difference between these two systems.

The gelation process of acidic system has been studied, focusing on the formation of multi-layers and orientation in the hydrogel^5^,^20^. In order to see the whole picture, understanding of alkaline system should be obtained as the missing puzzle piece. Previous works had proposed and discussed hypothesis of gelation mechanism^10^,^11^,^21^, but the gelation process of alkaline system requires more illumination in addition to current circumstantial evidence. Due to the particularity of alkaline system, the choice of characterization techniques were restricted. Techniques like SEM and TEM suffers from the sacrifice of native state of hydrogel and interferences of crystal formation of LiOH and urea^2^,^22^. On the other hand, techniques like light scattering provide information of native state but failed to give direct observation. Furthermore, quick data acquisition is also required to capture the whole gelation process. When taking these factors into account, fluorescent imaging is an ideal strategy^21^. In the present work, dynamic *in-situ* fluorescent imaging was employed to realise real-time observation of the gelation process. Together with other *in-situ* investigations, the differences between alkaline system and acidic system were studied, by analysing the gelation mechanism, process, and structural characters.

## Results

### Difference in mechanical properties of hydrogels via alkaline and acidic solvent

**Figure 1:**
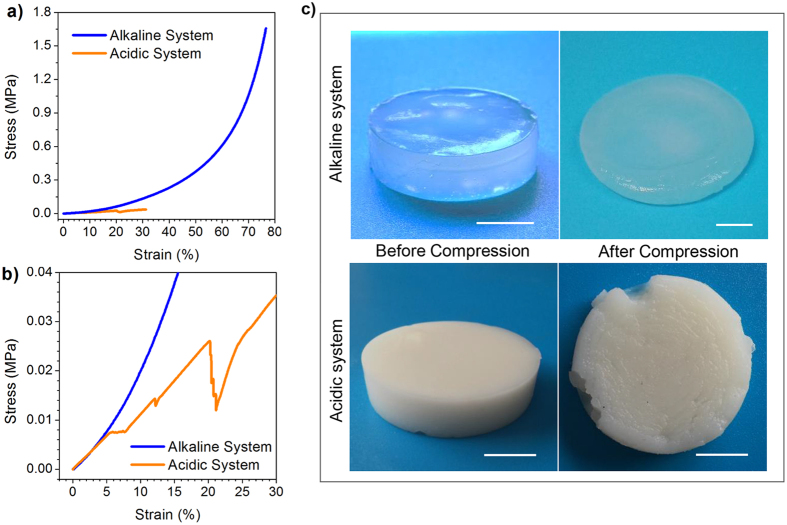
Difference in mechanical properties of CS gel via alkaline and acidic solvent. (**a**) compressive stress-stain curves; (**b**) compressive stress-stain curves at low strain range, *c*(CS) = 3 *wt.*%; (**c**) digital photographs of CS hydrogel samples before and after compression tests; scare bar:1 cm.

When comparing CS hydrogels via alkaline system and acidic system, the first impression would certainly be the remarkable difference in mechanical property, as demonstrated in Fig. 1a. The improvement of hardness had been reported as one of the advantages of CS hydrogel via alkaline system^11^. But when focusing on their mechanical behavior at low compressive strain, unlike reported in previous study, CS hydrogels via alkaline and via acidic solvent showed comparable compressive modulus (Fig. 1b). This result was actually very reasonable because when it comes to the mechanical property of CS hydrogel via acidic system, the *c*(OH^−^) (concentration of OH^−^) of coagulation bath must be considered as a parameter. In the present work, the coagulation bath had high concentration of OH^−^ (*c*(OH^−^) = 10 *wt.*%). When *c*(CS) (concentration of CS in the CS solution) is held constant, coagulation bath with higher *c*(OH^−^) will create tougher hydrogel, which is determined by the relationship between gelation rate and disentanglement of CS chains^5^. This indicated that the improvement of modulus may not be the absolute and overwhelming superiority of alkaline system CS hydrogel. However, with the increase of strain, the curve of acidic system hydrogel indicated the occurrence of yield and break (Fig. 1b), with cracks appearing in the hydrogel (Fig. 1c). But CS hydrogel via alkali-urea solvent deformed greatly during the compression without breaking even at high strain (Fig. 1c), and showed ultimate stress over 50 times higher than its counterpart via acidic solvent. The result above indicated that the high ultimate strength and energy absorption before breaking are the intrinsic advantages of CS hydrogel via alkaline system.

### Gelation process of hydrogels via alkaline and acidic solvent

**Figure 2:**
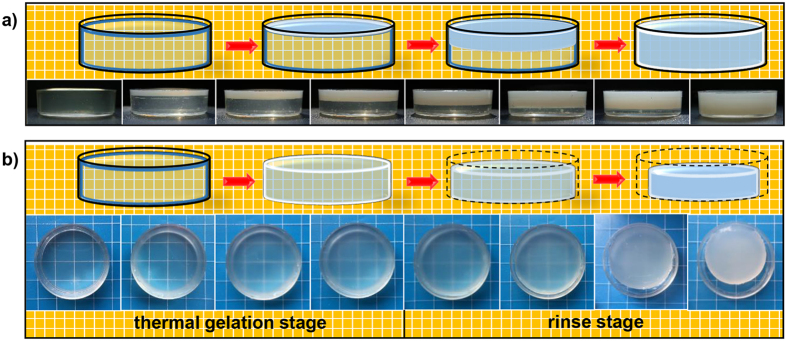
Schematic illustration and digital photographs of the gelation process of CS hydrogel. (**a**) via acidic solvent; (**b**) via alkaline solvent.

The differences between CS hydrogels via two systems, such as the difference in mechanical properties, are determined by the structure of hydrogel. To be more fundamental, they were determined by the gelation mechanism and process. For acidic system, the nature of gelation mechanism is the deprotonation and entanglement of CS macromolecules. While for alkaline CS system, evolution of intermolecular H-bonds interactions is the essence of the gelation mechanism^21^. The difference in gelation mechanism leads to the great difference in gelation process (Fig. 2) despite of the similarities between their procedures (Supplementary Fig. S1).

**Figure 3:**
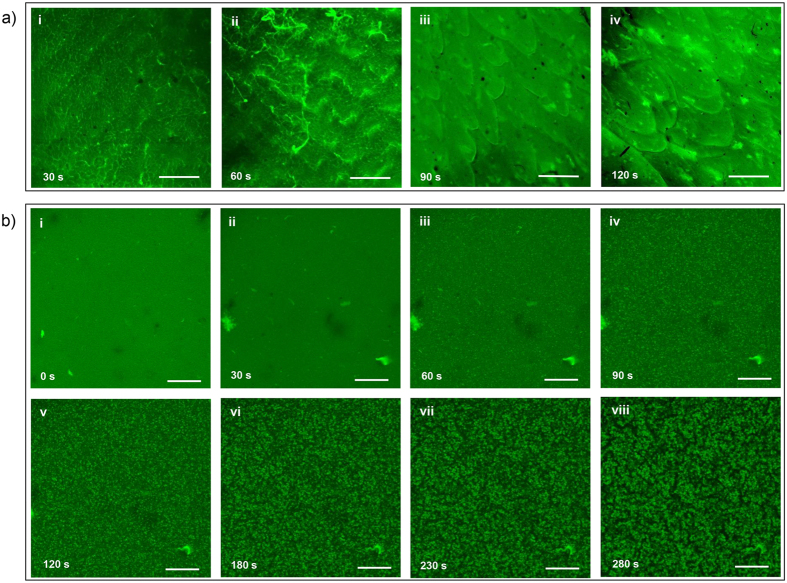
Observation of the gelation process by confocal laser scanning fluorescence microscope (CLSM). (**a**) via acidic solvent, scale bar-50 μm; (**b**) via alkaline solvent, scale bar-100 μm; *c*(CS) = 2 *wt*.%.

In acidic system, the gelation process is promoted by the diffusion of OH^−^ from the coagulation bath. Instead of forming cross-links in the whole system simultaneously, there exists a distinct sol-gel interface. It has been reported in our previous study, the gelation process of this system possessed a layer-wise character due to the equipotential surface of *c*(OH^−^). This character brings spatiotemporal sequence to the system, which endows orientation along the direction of OH^−^ diffusion^5^. The formation of acidic system CS hydrogel was shown in native wet state (Fig. 3a). It can be observed that the cross section of CS hydrogel was smooth at first. However, with the increase of gelation time and gel thickness, the cross section became more and more rough and eventually showed fibrous structure. Significantly different from the acidic system, alkaline system evolves in its entirety. The gelation process of alkaline system includes two stages, which could be summarized as the thermal gelation stage and the rinse stage^21^.

In order to explore the gelation process of alkaline system from a microcosmic level, dynamic *in-situ* fluorescent imaging was performed. Fluorescein iso-thiocyanate (FITC) was employed in the fluorescence labelling of CS. The resultant fluorescein iso-thiocyanate-labelled CS (FITC-CS) showed good fluorescence property (Supplementary Fig. S2) and good solubility in the alkaline solvent (Supplementary Fig. S3a,b). Then the solution could be used to prepare FITC-CS hydrogel following exactly the same procedures (Supplementary Fig. S3). Results indicated that the gelation process was not interfered by the introduction of FITC fluorogens (Supplementary Figs S4 and S5). The gelation process was visualized and presented in Fig. 3b and Video S1. No specific patterns were observed in fluorescent image which corresponded to the solution (Fig. 3b-i). But when gelation process was initiated, some bright spots started to appear (Fig. 3b-ii,iii). The amount of bright spots increased as time went on, and gradually became connected to each other (Fig. 3b-iv to vi).

This process could be understood in terms of the formation and percolation of aggregates. The percolation of aggregates in alkaline system shared similarity with typical thermogels (*e.g.* PLGA-PEG-PLGA)^23^ But the essential difference is that the unmodified CS itself does not possess thermosensitivity at all. The thermosensitivity of alkaline system originates from the evolution of intermolecular interactions between CS macromolecules and other system components. Aggregates of CS macromolecules existed in the solution with LiOH and urea acting as protective covers^2^. In the intermolecular interactions regarding dissolution and gelation, OH^−^ played the dominant role with the support of urea. With the increase of temperature, CS tends to form inter-/intramolecular H-bonds among CS rather than forming effective interaction with OH^−^ (Supplementary Fig. S8)^21^. Thus aggregation of macromolecules takes place due to self-association tendency, and the amount of aggregations increased with the gradual weakening of protection. This process terminated when the new equilibrium was established at the elevated temperature, and the system transferred from a polymer solution to a 3D network. Such network structure can also be observed in alkaline system with different *c*(CS) (Supplementary Fig. S6).

Alkaline system showed evident shrinkage during gelation process, especially in the rinse stage (Supplementary Fig. S7). The driving force of rinse stage is the continuous evolution of system components, and further evolution of structure happened at the same time. It has been proven that the volume decrease was not a simple shrinkage due to the loss of solvent components^21^. As a matter of fact, the underlying reason is the solvophilic/solvophobic interactions, which could be interpreted by progressive loss of solubility (demonstrated in Supplementary Fig. S2d). During the entire gelation process, solvent quality decreases consecutively, leading to the volume decrease of hydrogel and structural change all along^24^. In contrast to this phenomenon, the acidic system showed little volume change during gelation process. Due to the fast neutralization reaction, CS chains in the acidic system turned into entangled hydrogel rapidly. So the rinse procedure afterward only removed the OH^−^ introduced by coagulation bath, and did not alter the balance of solvophobic and solvophilic interactions.

### Different crystallinity of hydrogels via alkaline and acidic solvent

**Figure 4:**
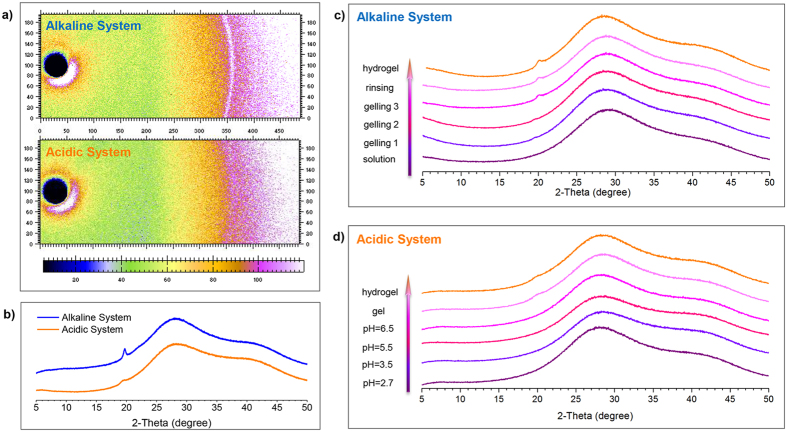
Difference in crystalline of CS gel via alkaline and acidic solvent. (**a**) WAXS patterns of CS hydrogel via alkaline and acidic solution, *c*(CS) = 3 *wt.*%; (**b**) XRD diffraction profiles of CS hydrogel via alkaline and acidic solution, *c*(CS) = 3 *wt.*%; (**c**) evolution of crystalline in CS alkaline system during gelation process, *c*(CS) = 2 *wt.*%; (**d**) evolution of crystalline in CS acidic system during gelation process, *c*(CS) = 2 *wt.*%.

The difference in gelation process can lead to the difference of crystallinity. In hydrogels, crystalline is very important as one form of physical junction. The investigation of crystalline in CS hydrogels via both systems was performed in native state by X-ray diffraction (Fig. 4). A bright diffraction ring can be observed in the WAXS pattern of alkaline system, but not observed in the acidic system (Fig. 4a). The X-ray diffraction profiles of alkaline system and acidic system samples were shown in Fig. 4b. The broad peak belonged to liquid water^25^. The profiles of both alkaline system and acidic system samples showed a peak at 2*θ *= 20°, corresponding to the 110 reflection of CS crystalline^26^. The degree of crystallinity and the size of the crystals can be evaluated by the area and the full width at half maximum (FWHM) of diffraction peak, respectively^27^. For acidic system, the diffraction peak has small peak value and large FWHM, while the diffraction profile of alkaline system has higher peak value and smaller FWHM. The results indicated that alkaline system has advantage over acidic system in both the degree of crystallinity and the size of the crystals. This result is repeatable and can be observed in systems with different *c*(CS) (Supplementary Fig. S9). To account for this difference, the time range for crystalline development in gelation process should be understood. For alkaline system, the increase of peak intensity lasted the whole thermal gelation process (Fig. 4c)^21^, which was a long and mild process compared to acidic system. The evolution of crystalline in CS acidic solution was studied by simulating the sol-gel transition of CS in acidic system (Fig. 4d). During the gelation process of acidic solution, when the gelation front reached a certain point in the system, this position went through OH^−^ diffusion and the increase of pH. Results indicated that acidic CS solution showed no diffraction peak of CS crystalline, and such peak did not appear with the increase of pH. When pH = 6.5, the system got close to sol-gel transition and the sample was a solution with very high viscosity. But the diffraction peak still did not appear. After the system turned to hydrogel, a small broad peak could be observed at 2θ = 20°, and the peak showed no further development after rinse procedure. This indicated the formation of crystalline in acidic system was very rapid, which only occurred upon sol-gel transition. Moreover, the sol-gel transition happened at the gelation front, which moved forward at high speed (in the magnitude of several micrometres per second)^5^, making it impossible for the crystalline to fully develop.

### Hierarchical structure of hydrogels via alkaline and acidic solvent

**Figure 5:**
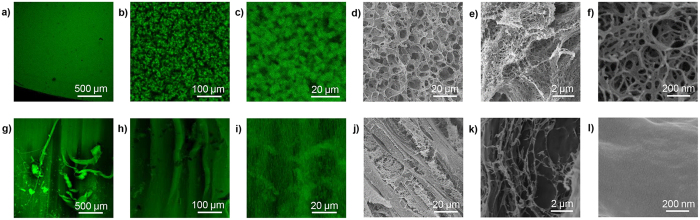
Hierarchical structure of CS hydrogel. (**a**–**f**) via alkaline solvent; (**g–l**) via acidic solvent, *c*(CS) = 2 *wt.*%. (**a–c,g–i**) were confocal laser scanning fluorescence microscope images; (**d–f,j–l**) were SEM images.

**Figure 6:**
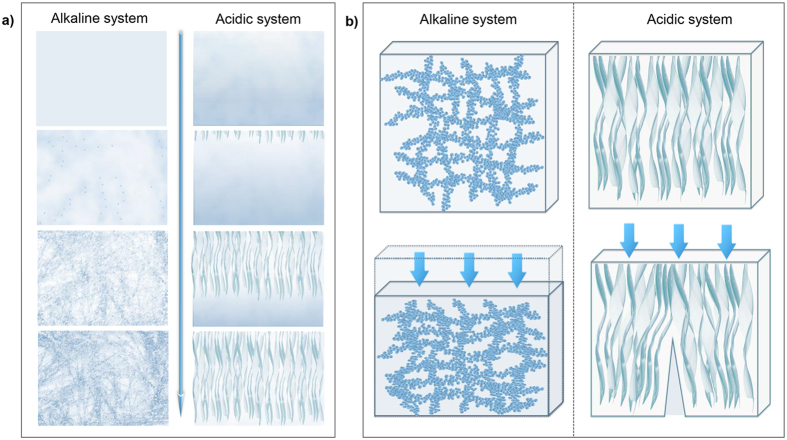
Schematic illustration of structural characters of CS hydrogel via alkaline and acidic solvent. (**a**) gelation process and structure evolution of CS hydrogel. (**b**) CS hydrogel during compression.

The difference in gelation process ultimately leads to the difference in structure. CS hydrogel morphology was shown on different hierarchical structural levels (Fig. 5). In present work, results showed that CS hydrogel via acidic solvent had orientated fibrous structure. The structure of one fiber was rather compact while larger voids existed among those fibers. CS hydrogel coagulated by bath with low *c*(OH^−^) had very low modulus and randomly located voids, which would not be discussed further. CS hydrogel via alkaline solvent possesses hierarchical organization as a uniform network structure. Network structure even existed in nanoscale for CS hydrogel via alkaline system, which was very different from the acidic system. The gelation process and structure of alkaline and acidic system were summarized and schematically illustrated in Fig. 6a. The gelation process of both acidic and alkaline systems started with homogeneous CS solution. In acidic system, phase separation happened in the primary hydrogel layer. Then during the layer-wised gelation process, macromolecules in the gelation front were inclined to rearrange below CS-rich zones of the previous layer^5^, and finally forming oriented fibrous structure. In alkaline system, aggregates formed uniformly in the system and connected to be a 3D network. Then in the rinse stage, structure evolved based on the already formed network. The evolution was more localized in this stage and numerous inter-/intramolecular hydrogen bonds were formed among macromolecules. Finally, uniform network came into being.

So the breaking pattern of CS hydrogel was analyzed as follow (Fig. 6b). For acidic system CS hydrogel, the large voids caused dislocation of fibers during compression, and the dislocation finally developed to be cracks in the material. While for alkaline system, the uniform network structure created uniform distribution of loading, which was favored in the improvement of mechanical strength^28^. Besides, uniform network reduced structural defects in the hydrogel. As a result, CS hydrogel via alkaline system did not break at low strain, and the structure became more and more compact. So the gradient of stress-strain curve kept increasing as shown in Fig. 1. Moreover, the alkaline system CS hydrogel contains more crystalline domains, acting as knots in the gel network. The presence of crystalline knots improved dimensional stability of the gel and induced elastic properties^25^.

## Discussion

After understanding the gelation process, characters of the two systems should be taken into consideration when designing CS hydrogel materials to suit various needs and applications. Because these characters not only determined the choice of modulation parameters, but also determined the structure and function of the resultant materials. To be more specific, the acidic system is desirable in the following cases. (1) The modulation parameter is relevant to the change of pH value, *e.g.* diffusion of ions (especially OH^−^), or the pH change caused by electrical signals. (2) The material requires sophisticated organization like multi-layers or oriented fibres. On the other hand, the alkaline system is desirable in the following cases. (1) Temperature is the modulation parameter. (2) The ultimate strength and toughness is priority. (3) The material requires uniform or isotropic structure. Moreover, the two systems can be utilised together, to create CS hydrogel that combines the advantage of mechanical strength and delicacy of structure. In addition to the preparation of pure CS materials, these systems could also be used to fabricate CS composite materials. CS composite materials could be prepared by introducing other substances (such as metal ions or certain reinforcements). Some gelation parameters may vary, but the gelation mechanisms of macromolecules were essentially the same as the pure CS systems.

In summary, differences between CS alkaline system and acidic system were studied. Finally, the reasons behind these differences were explained, accompanied by discussion about the design strategies based on these two systems.

## Methods

### Materials

Chitosan was prepared from chitin by heterogeneous N-deacetylation. The chitin was from Zhejiang Gold Shell Pharmaceutical Co., Ltd. The parameters of CS were as follows: the average viscosity molecular weight was 1.12 × 10^6^ Da, and the degree of deacetylation was 90.4%. Acetic acid, LiOH·H_2_O, NaOH, and urea were of analytical reagent grade (Sinopharm Chemical Reagent Co., Ltd). Fluorescein iso-thiocyanate (FITC) was purchased from Sigma Chemical Company. Fluorescein iso-thiocyanate-labelled CS (FITC-CS) was prepared following the preparation procedure reported in literature^29^.

### Formulation of CS solution via acidic and alkaline systems

In the present work, *c*(CS) was defined as the weight percentage of CS in the CS solution. For the preparation of CS hydrogel via acidic system, the solvent was 2 *vol.*% acetic acid aqueous solution. For the preparation of CS hydrogel via alkaline system, the solvent was an aqueous solution with weight percentage of LiOH-4.8 *wt.*% and urea-8.0 *wt.*%. CS solution samples with different *c*(CS) were prepared for different tests. For CS solution with *c*(CS) = *x wt.*%, *x* g CS powder was added in (100-*x*) g solvent.

### Mechanical tests

A universal materials testing machine (Instron, 5543A) was used to perform compression tests on cylinder CS hydrogel sample. The strain rate was 2% min^−1^ and the tests were performed at room temperature. In this section, the diameter and depth of the mold was 33 mm and 4 mm, respectively.

### Observation of hydrogel formation via acidic solvent

Firstly, gelation process was started. After a short period of time, the gelation process was terminated by separating hydrogel already formed from unreacted FITC-CS solution. The separated hydrogel was immediately washed to remove the residual FITC-CS solution. This step was performed meticulously without touching the surfaces of separated hydrogel. The thickness of hydrogel is related to the gelation time. When the gelation process was terminated at different gelation time (30 s, 60, 90 s, 120 s), the observed surface reflected the cross section of hydrogel with different thickness, *i.e.* different distance to the primary hydrogel layer. Experiment in this section was equivalent to prepare slices of hydrogel on the cross section.

### *In-situ* observation of hydrogel formation via alkaline solvent

Confocal laser scanning fluorescence microscope (CLSM) was employed in the *in-situ* observation of hydrogel formation (Leica TCS SP5). FITC-CS alkaline aqueous solution (*c*(FITC-CS) = 2 *wt.*%) was prepared, then filled in a quartz culture dish. The FITC-CS alkaline solution was −4 °C originally. Heat needed in the thermal gelation stage was from the heat source (predetermined to be 40 °C). Timing began as soon as the sample contacted the heat source.

### Morphology observation

CS hydrogel samples were studied by scanning electron microscopy (SEM) (HITACHI S-4800 SEM). For SEM observation, CS hydrogels were freeze-dried and then gold-sprayed for conductance. CS hydrogels were also studied by CLSM; hydrogels were prepared with FITC-CS and kept wet during observation.

### X-ray diffraction

Wide-angle X-ray diffraction tests were performed on Ultima IV analytical instrumentation (Rigaku Corporation) at room temperature. Ni-filtered Cu Kα radiation was used and scanned in the 2*θ* range of 5–50° at the rate of 2 degree (2*θ*) s^−1^. A tailor-made specimen holder (thickness: 2 mm) was used. The evolution of crystalline in CS alkaline solution was studied by the following experiment. In the thermal gelation stage, the temperature was predetermined to be 60 °C. The sample was heated for 5 min (denoted as gelling 1), 10 min (denoted as gelling 2) and 15 min (denoted as gelling 3) before every measurement, respectively. In the rinse stage, the gel was washed with 500 mL deionized water for 60 min (denoted as rinsing), and then rinsed till LiOH and urea was completely removed (denoted as hydrogel). The evolution of crystalline in CS acidic solution was studied by the following experiment. First, CS acidic solution was prepared according to procedure described above, and the pH was determined to be 2.7. Then NaOH was added to modulate the pH, and samples with different pH were prepared (pH = 3.5, pH = 5.5, pH = 6.5). The X-ray diffraction profiles of CS gel (without washing) and hydrogel (after washing procedure) were also collected.

## Additional Information

**How to cite this article**: Nie, J. *et al*. Difference between Chitosan Hydrogels via Alkaline and Acidic Solvent Systems. *Sci. Rep.* **6**, 36053; doi: 10.1038/srep36053 (2016).

**Publisher’s note:** Springer Nature remains neutral with regard to jurisdictional claims in published maps and institutional affiliations.

## Supplementary Material

Supplementary Video S1

Supplementary Information
