# Supplementary material for: Difference between Chitosan Hydrogels via Alkaline and Acidic Solvent Systems
Source: Sci Rep. 2016 Oct 27;6:36053. doi: 10.1038/srep36053 (PMC5081545; doi:10.1038/srep36053)
Supplement: Supplementary Information [file srep36053-s2.pdf]

Supplementary Information

Difference between Chitosan Hydrogels via Alkaline and Acidic Solvent Systems

Jingyi Nie,[1, 2] Zhengke Wang, [1, 2] * Qiaoling Hu [1, 2]

[1] MoE Key Laboratory of Macromolecular Synthesis and Functionalization, Department of Polymer Science and Engineering, Zhejiang University, Hangzhou, China. [2] Key Laboratory of Adsorption and Separation Materials & Technologies of Zhejiang Province, Hangzhou, China.

Correspondence and requests for materials should be addressed to Z.K.W. (email: wangzk@zju.edu.cn).

For the preparation of CS hydrogel via acidic system, CS solution with desired concentration, *i.e.* c(CS), was prepared by dissolving CS powder in 2 *vol.*% acetic acid aqueous solution. Coagulation bath was 10 *wt.*% NaOH aqueous solution. The volume of CS solution for one hydrogel sample varied slightly with different kinds of molds used in this study, and was in the range of 2-10 mL. For one hydrogel sample, the volume of coagulation bath was at least 1000 mL. The volume of coagulation bath was much larger than that of CS solution, so the $c(OH^-)$ could be considered as constant. A single opening mold was used to fabricate the hydrogel. CS solution was filled in the mold (Supplementary Fig. S1a) and immersed in the coagulation bath (Supplementary Fig. S1b, c) till the gelation was completed. After that, resultant CS gel was unloaded from the mold (Supplementary Fig. S1d). Then the CS gel was rinsed repeatedly to be neutral with deionized water (Supplementary Fig. S1e). The rinse procedure was monitored by a pH meter (PHS-3C, INESA Scientific Instrument Co., Ltd). The procedure ended when the pH of rinsing bath reached the pH of deionized water and did not increase anymore.

For the preparation of CS hydrogel via alkaline system, the solvent was prepared as follows. $LiOH{\cdot}H_2O$ and urea were dissolved in deionized water to form a transparent solution with weight percentage of LiOH-4.8 *wt.*% and urea-8.0 *wt.*%, respectively. A desired amount of CS powder was mixed in the solvent. The mixture was then treated by a freeze (-40 ^{o}C) - thawing (20 ^{o}C) process three times to get transparent CS LiOH-urea solution. The solution was filled in a single opening mold to fabricate the hydrogel (Supplementary Fig. S1f). The solution was held for 30 min

in a 40 °C thermostatic water bath (Supplementary Fig. S1g) and turned to CS gel containing LiOH and urea (Supplementary Fig. S1h), which corresponded to the thermal gelation stage. After the mold was unloaded (Supplementary Fig. S1i), the resultant gel was then fully rinsed in deionized water till the full removal of LiOH and urea (Supplementary Fig. S1j), which corresponded to the rinse stage.

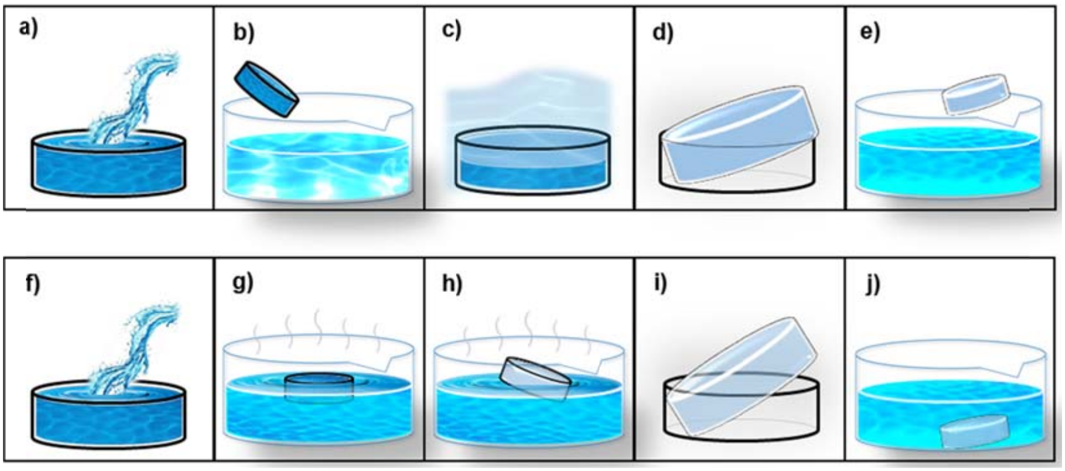

Supplementary Figure 1 | Schematic illustration of the preparation of CS hydrogel via acidic and alkaline systems, respectively. a-e) Preparation via acidic system: (a) the acidic CS solution was filled in the mold; (b) the mold was immersed in the coagulation bath; (c) gelation process of acidic CS solution; (d) the gel was unloaded from the mold; (e) the gel was washed by deionized water. (f-j) Preparation via alkaline system: (f) the alkaline CS solution was filled in the mold; (g) thermal gelation was induced by hot water bath; (h) thermal gelation finished; (i) the gel was unloaded from the mold; (j) removal of LiOH and urea.

The degree of labeling (DL, mol %) of FITC-CS was 0.09 %.[1] FITC-CS LiOH-urea solution was prepared according to the freeze-thawing process described above. A UV-vis spectrophotometer was used to obtain the ultraviolet (UV) absorption

spectra of FITC-CS solution with a quartz cell. Fluorescent (FL) spectra of the FITC-CS LiOH-urea system were measured on a Perkin-Elmer LS 55 spectrofluorometer.

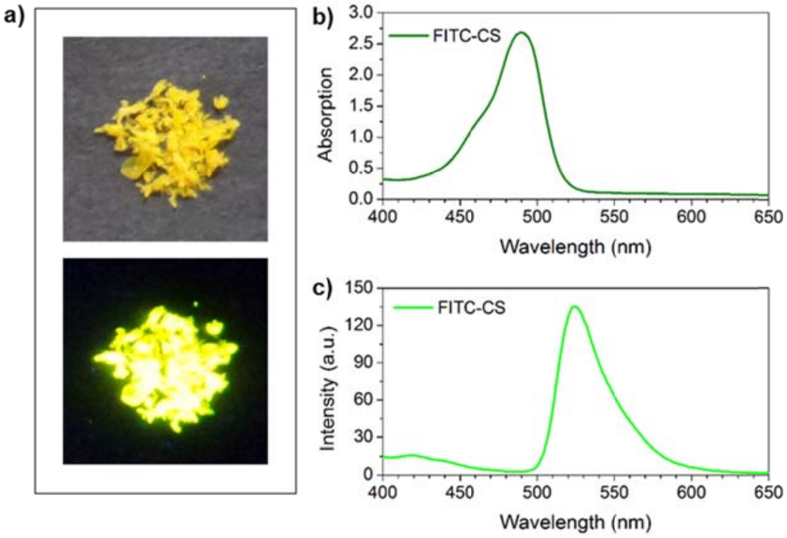

Supplementary Figure 2 | FITC-CS samples. (a) FITC-CS powder, the fluorescent photographs were taken under UV illumination (365 nm); (b) ultraviolet absorption spectra of FITC-CS alkaline solution; (c) fluorescent spectra of FITC-CS alkaline solution.

FITC-CS can be fabricated into hydrogel by the same preparation procedures as shown in Supplementary Fig. S3. The concentration of FITC-CS used in this section was 2 *wt.*%.

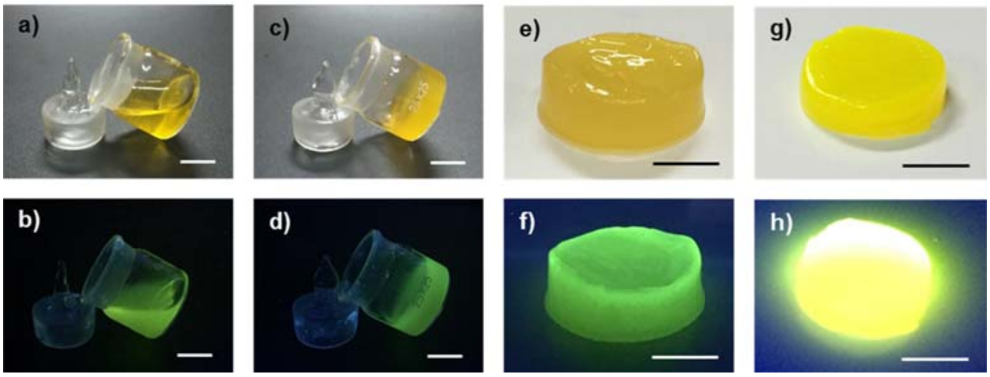

Supplementary Figure 3 | Digital photographs demonstrating the formation of

hydrogel with FITC-CS. (a, b) solution of FITC-CS; (c, d) FITC-CS gel after thermal gelation; (e, f) unloaded FITC-CS gel after thermal gelation; (g, h) FITC-CS hydrogel after complete removal of LiOH and urea; scale bar -5.0 mm. The fluorescent photographs were taken under UV illumination (365 nm).

FITC-CS sample formed a transparent solution (Supplementary Fig. S3a, b). This indicated that, with proper DL, the introduction of FITC fluorogens did not exert much interference on the solubility of CS. Volume shrinkage can be observed during the rinse stage of FITC-CS hydrogel, which was in good accordance with behavior of pure CS hydrogel. Besides, the hydrogel sample showed stronger fluorescence than gel containing LiOH and urea. This is because the FITC fluorogens were responsive to the change of pH.[2]

The sol-gel transition of CS LiOH-urea and FITC-CS LiOH-urea aqueous solutions at different concentrations or temperatures were determined by the vial-inverting method. 1.0 mL of solution was added into a 2 mL vial. The vials were immersed in a water bath with constant temperature. The sample was equilibrated at 4 °C for 2 h before measurements. If no flow was noticed in 30 s after the vial was inverted, the aqueous system at this concentration and temperature was judged as in a gel state. The well matched gelation time of CS and FITC-CS indicated that (Supplementary Fig. S4), with proper DL, the introduction of FITC fluorogens did not exert much interference on the gelation process of CS. The X-ray diffraction profile of FITC-CS LiOH-urea gel *in-situ* formed were collected. The conditions were identical to the tests of CS LiOH-urea system. The result (Supplementary Fig. S5) indicated

that the crystalline formation in gelation process was not affected by the introduction of FITC fluorogens either.

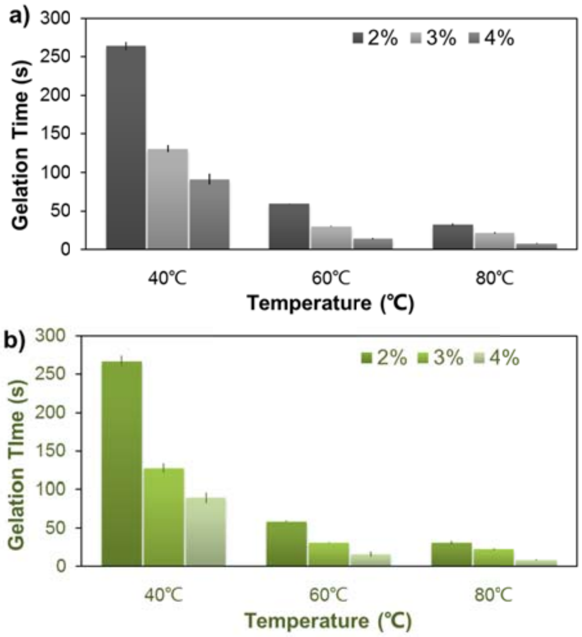

Supplementary Figure 4 | Gelation time of CS and FITC-CS. Gelation time of CS (a) and FITC-CS (b) respectively, under different temperature and concentration.

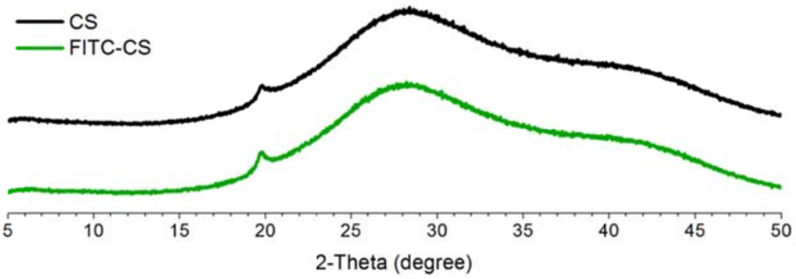

Supplementary Figure 5 | XRD patterns of FITC-CS LiOH-urea gel and CS LiOH-urea gel *in-situ* formed, 2 *wt*.%.

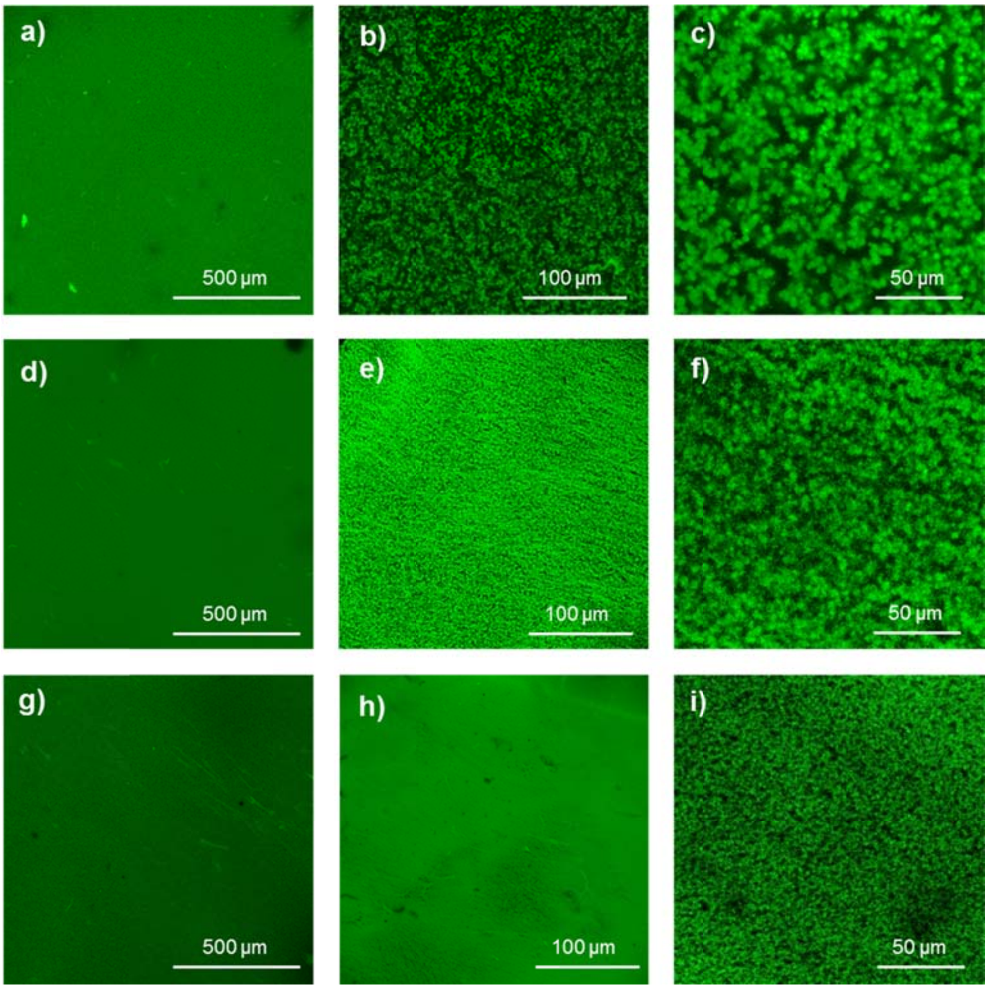

Supplementary Figure 6 | CLSM images of FITC-CS hydrogel with different concentration. (a-c) 2 *wt.*%; (d-f) 3 *wt.*%; (g-i) 4 *wt.*%.

The 3D network structure can also be observed with higher *c*(CS) (Supplementary Fig. S6). With the increase of *c*(CS), the network was more uniform and compact. The possible reasons were demonstrated as follows. When other parameters were held constant, the concentration of aggregates in the system increase with the rise of *c*(CS), thus increasing the probability of aggregates connection. Moreover, it has been reported that the size of aggregate increased with time after triggered by heat. So with higher concentration of aggregates, the connection with each other can be realized faster with smaller aggregate size. The more uniform and

compact network structure explained the evident improvement of modulus and ultimate strength with rise of *c*(CS) in previous study.[3]

The tests of volume shrinking behaviors of CS gel were carried out by measuring the diameter change of hydrogel discs.[4] For CS hydrogel via acidic system, the mold was unloaded after the gelation front advanced to the bottom of the mold, and then the gel was rinsed for 10 min in 200 mL deionized water before every measurement. For CS hydrogel via alkaline system, the mold was unloaded after enough thermal gelation time, and then the gel was also rinsed for 10 min in 200 mL deionized water before every measurement. The results were shown in Supplementary Fig. S7.

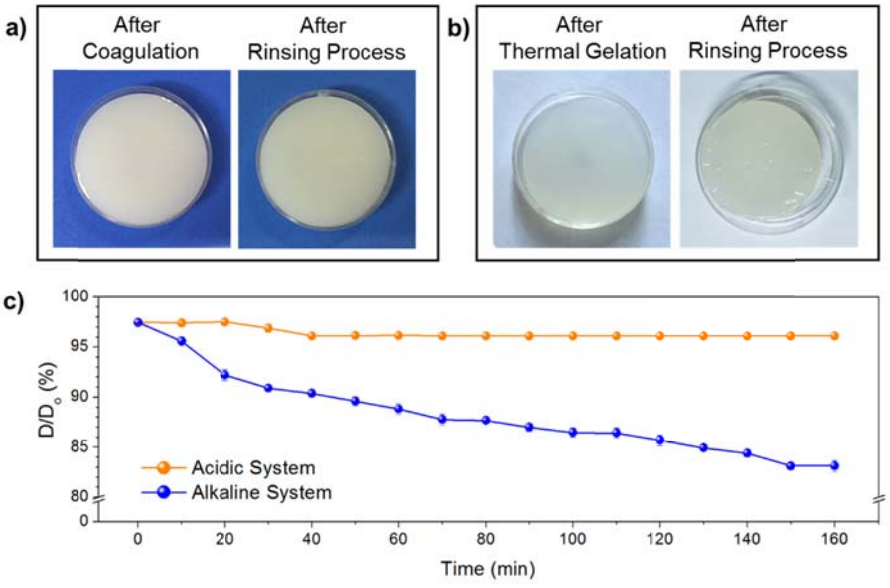

Supplementary Figure 7 | Images and the dimension shrinkage rate of CS gel. (a) via acidic system; (b) via alkaline system; (c) dimension shrinkage rate of hydrogel with time. D_0 is the diameter of mold and D is the diameter of gel.

The results above could be interpreted by the Flory-Huggins χ parameter. Only in this case, the solvent was very special. The χ parameter reflected the change of

interaction energy upon the mixing of macromolecules and solvent, thus reflected the solubility of solvent. According to polymer physics theory, in good solvent the χ parameter has lower value and the chain of polymer is extended. In poor solvent with higher value of χ parameter, polymer coils contracted, leading to polymer collapse and possible phase separation.[5] As reported in our previous study,[6] the intermolecular interactions in the system can be described as the overall reaction of two competitive interactions. Reaction 1 is the formation of H-bonds between OH^- and CS (Supplementary Fig. 8a), and reaction 2 is the formation of inter-/intramolecular H-bonds among CS. When the system temperature increased, the frequency of detach and re-attach of OH^- with CS became higher. Meanwhile, the frequency of formation and breaking of H-bonds was also increased with the rise of system temperature. However, due to the limited mobility of macromolecules (compared to OH^-), the increase of the former frequency was greater than the latter one. So reaction 2 is less susceptible to heat than reaction 1. In the thermal gelation process, the formation of inter-/intramolecular H-bonds among CS gradually became dominant, and the effective interaction with OH^- decreased (Supplementary Fig. 8b). Thus the solubility decreased and the χ parameter increased, because it required more energy to mix the solute and the solvent. In the rinse stage, *c*(LiOH) and *c*(urea) gradually decreased from original value to zero. The change of *c*(LiOH) and *c*(urea) in the system largely promoted the reverse course of reaction 1 and the formation of inter-/intramolecular H-bonds among CS. Thus the solubility further decreased and the χ parameter kept increasing in this consecutive process. The χ parameter of increased during the entire

gelation process, with the volume of hydrogel decreased all along (demonstrated by quasi lattice model in Supplementary Fig. 8d). When the value of *c*(LiOH) and *c*(urea) of system became zero, the overall reaction reached an equilibrium, and the "solvent" held in the macromolecular network was pure water. The system became a hydrogel in equilibrium with water. So when CS hydrogel was kept in deionized water after the rinse stage, the equilibrium would not shift any more, thus there is no further swelling or shrinking.

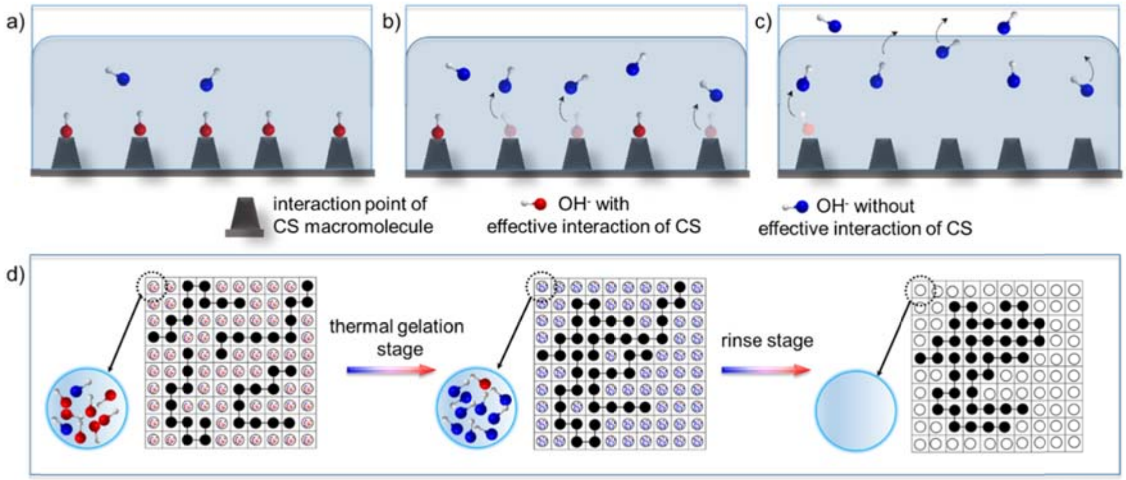

Supplementary Figure 8 |Schematic illustration of intermolecular interactions in CS LiOH-urea solution. (a) solution before thermal gelation; (b) thermal gelation stage; (c) in the rinse stage; d) schematic illustration of the intermolecular interaction by quasi lattice model.

Wide Angle X-ray Scattering (WAXS) experiment were performed using a small-angle diffractometer (Molecular Metrology SAXS system) with Cu Kα radiation from a sealed microfocus tube (MicroMax-002+S), two Göbel mirrors, and three-pinhole slits (generator powered at 45 kV and 0.9 mA). The scattering patterns were recorded by a 20×20 cm two-dimensional position sensitive wire detector that

was positioned 150 cm behind the sample. The result of WAXS was processed by Fit 2D software.

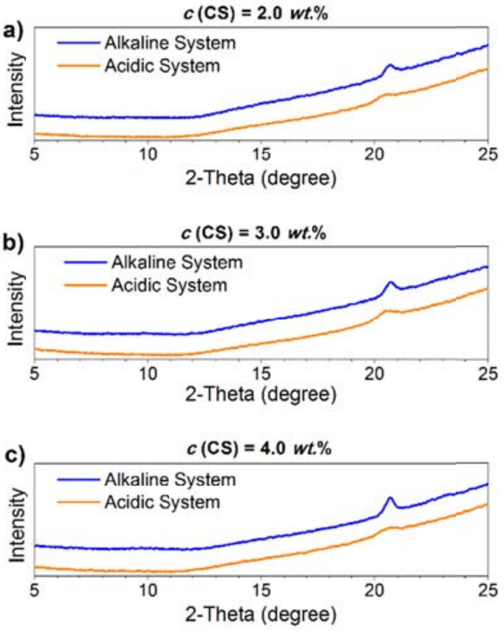

Supplementary Figure 9 | WAXS profiles of CS hydrogel via alkaline and acidic solution with different *c*(CS). (a) *c*(CS)=2 *wt.*%; (b) *c*(CS)=3 *wt.*%; (c) *c*(CS)=4 *wt.*%.

Supplementary Video | Thermal gelation of CS-LiOH-urea solution.

Reference

1 Wang, Z. *et al.* Long-term fluorescent cellular tracing by the aggregates of AIE bioconjugates. *Journal of the American Chemical Society* **135**, 8238-8245, (2013).

2 Ma, L. Y., Wang, H. Y., Xie, H. & Xu, L. X. A long lifetime chemical sensor: study on fluorescence property of fluorescein isothiocyanate and preparation of pH chemical sensor. *Spectrochim. Acta A.* **60**, 1865-1872, (2004).

3 Sun, Y. F., Li, Y. L., Nie, J. Y., Wang, Z. K. & Hu, Q. L. High-strength Chitosan Hydrogels Prepared from LiOH/Urea Solvent System. *Chem. Lett.* **42**, 838-840, (2013).

4 Mi, P. *et al.* A novel stimuli-responsive hydrogel for K^{+}-induced controlled-release. *Polymer* **51**, 1648-1653, (2010).

5 Michael Rubinstein, R. H. C. *Polymer Physics*. 173 (Oxford University Press, 2003).

6 Z. Wang, Z., Nie, J., Qin, W., Hu, Q. & Tang, B. Gelation process visualized by aggregation-induced emission fluorogens. *Nat. Commun.* **7**, 12033, (2016).
